# Energetic Conditions Promoting Top-Down Control of Prey by Predators

**DOI:** 10.1371/journal.pone.0029723

**Published:** 2011-12-27

**Authors:** Kristin N. Marshall, Timothy E. Essington

**Affiliations:** School of Aquatic and Fishery Sciences, University of Washington, Seattle, Washington, United States of America; University of Bristol, United Kingdom

## Abstract

Humans remove large amounts of biomass from natural ecosystems, and large bodied high trophic level animals are especially sensitive and vulnerable to exploitation. The effects of removing top-predators on food webs are often difficult to predict because of limited information on species interaction strengths. Here we used a three species predator-prey model to explore relationships between energetic properties of trophodynamic linkages and interaction strengths to provide heuristic rules that indicate observable energetic conditions that are most likely to lead to stable and strong top-down control of prey by predator species. We found that strong top-down interaction strengths resulted from low levels of energy flow from prey to predators. Strong interactions are more stable when they are a consequence of low per capita predation and when predators are subsidized by recruitment. Diet composition also affects stability, but the relationship depends on the form of the functional response. Our results imply that for generalist satiating predators, strong top-down control on prey is most likely for prey items that occupy a small portion of the diet and when density dependent recruitment is moderately high.

## Introduction

Ecological forecasting has emerged as a priority for ecologists over the last decade [1], given growing recognition of human impacts on ecosystems and the need for improved capability to predict their outcomes. Targeted removal of apex predators is one widespread anthropogenic impact affecting both terrestrial [2], [3], [4] and aquatic ecosystems [5], [6]. Predicting the nature and magnitude of direct and indirect effects that follow from these removals is limited by a paucity of detailed, site-specific information on interaction strengths in natural food webs. Yet, recent syntheses have revealed that top-down control of prey populations is both pervasive and highly variable among and between ecosystems [7], [8]. Therefore, tools to improve our ability to identify conditions under which direct effects of predator removals might be of greatest concern are in high demand.

At least two approaches have seen widespread use in measuring the effects of perturbations in predator populations in predator-prey systems. Experimental manipulations are arguably the most powerful and direct way to estimate this kind of interaction strength [9], [10], [11], [12]. Alternatively, time series and path analysis can also identify interaction strength when long term data on population densities are available [13], [14]. Both of these methods have been and will continue to be primary ways of estimation, but logistic or data requirements may preclude their use in many ecosystems. Large scale interaction strength experiments are logistically difficult in many environments such as coastal ocean ecosystems, and for species with large home ranges. Time series approaches or path analyses require long time series of population densities collected at a temporal frequency that is sufficient to distinguish between direct and indirect effects. Thus, these methods require monitoring data spanning many generations, and are likely to reveal impacts only after they have occurred.

Because these methods may not be practical for many ecosystems, applied ecologists often rely on other information to identify potential strong interactions between species. For instance, data on abundances, body sizes, consumption rates, and diet composition are commonly collected and can be used to develop energetic webs diagramming energy flow through trophic configurations (e.g. [15]). These depictions are useful in identifying important energy sources for individual species within a system, as well as energy sources for a whole system. Ecologists have noted, however, that strong energetic links are not generally indicative of strong dynamic interaction strengths [9], [16], [17], [18]. Thus, our ability to predict interaction strengths from energetic information remains limited.

This paper investigates the relationship between interaction strength and energetics to develop heuristic “rules of thumb” describing energetic conditions that are most likely to give rise to strong top-down interaction strengths. We approached this modeling problem from the same perspective facing applied ecologists who have a set of observations on biomasses of predator and prey populations and the energetic flux between them. Our work seeks to derive generalizations that might hold over many ecosystems, so we use simple abstract models (strategic, sensu [19]) rather than a detailed model constructed around a specific ecosystem. To this end, we developed a generalized three-species predator-prey model and used this model to explore the patterns of interaction strength that emerged from alternative energetic conditions.

Theoretical research coupling energetics with dynamic predator-prey models over the last decade has demonstrated a common motif that provides stability in food webs consists of one strong interaction coupled with multiple weak interactions [20], [21]. Our work extends previous research in this area in two important ways. First, most theoretical work has defined interaction strength as a purely per-capita effect [21], i.e. the per-capita effect of a change in abundance of one species on another (but see [22] for an exception). We maintain that it is equally important from a management and conservation perspective to understand the net effect of widespread depletion of predators on prey species. Second, our goal is to use simple models to reveal generalized patterns about energetic configurations - which might be identified based on relatively common types of available data - that give rise to stable and strong top-down regulation of prey. These generalizations comprise heuristics that can guide more detailed study and forecasts about effects of predator removals on prey populations in an applied setting.

## Methods

Our modeling perspective is fundamentally different from those commonly employed in theoretical derivations of predator-prey models. Most derivations specify underlying biological processes for each population and then use those assumptions to define steady state conditions. From an ecological forecasting perspective, the path of information flow is reversed: the steady state conditions are observed and some information about the nature of the underlying processes is inferred so that predictions about the effects of species removals can be made [23]. We explored what information about interaction strength can be abstracted from knowledge of predator–prey population biomasses and the energy flux between them. Our ultimate goal was to identify processes that lead to more stable predator-prey interactions, with the underlying assumption that more stable configurations are more likely to persist and thereby be more prevalent in nature.

Predicting strong top-down control first requires defining what constitutes a strong interaction. Here, we refer to the effect of removal of a predator on equilibrium prey response as “interaction strength,” which is analogous to “absolute prey response” as defined by Berlow et al. [24]. Because experimental manipulation studies routinely use this metric, it provides a way to couple our analytical model with experimental data or hypothetical scenarios of predator removal. Multiple aspects of our model could be considered interaction strength metrics (e.g. consumption rates, functional responses, and elements of the community matrix). However, we focus on how various parts of our model lead to strong effects of perturbations in predator populations, as that is often most meaningful in an applied management setting.

### Model Structure

We developed a simple predator-prey model based on commonly used functions that relate predator and prey population dynamics. Model parameters were then related to energetic properties that might be observed, and we explored how those properties constrain the underlying behavior of the system. Finally, we examined the range of possible predator-prey dynamics that may have produced observed steady state conditions.

In our model, we allow the predator population's total biomass, *P*, to be composed of three alternative energy sources. The first two sources are the consumption of a preferred and alternative prey. The third source is reproduction, whereby a predator's offspring “recruit” to the adult stage (following growth and trophic ontogeny). Thus, predator biomass greatly exceeds that invested in offspring, and offspring are trophically de-coupled from the predator-prey dynamics occurring during the adult stage.

The biomass dynamics of predator and each prey species (*B*_1_ and *B*_2_) include these three energy fluxes
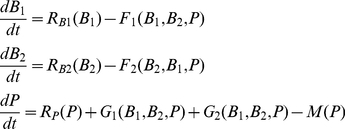
(1)


The functions 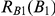 and 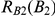
describe the net production rate of each prey species in the absence of predation. The functions 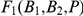
and 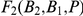 describe predator consumption of prey biomass. Note that the model implicitly assumes no direct interaction between the two prey species. For predators the functions denoted *G* describe energy gained from consumption: 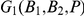 describes the energy gained from consuming prey species 1, while 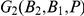
describes predator's energy gain from consuming an alternative prey. *R_P_*(*P*) describes the recruitment of predator offspring that have completed ontogeny into the adult stage. Lastly, the function *M*(*P*) describes biomass lost through mortality.

This abstract model can be used to identify how model components relate to real-world, observable quantities. Namely, the observed steady state quantities P* and *B*_1_* and *B*_2_* tell us something about the values of the model functions because these values are constrained to produce *dB*_1_/*dt*  = *dB*_2_/*dt*  = *dP*/*dt*  = 0. Also, the observed total consumption rate of each prey by predators equals the value of the function *F*_1_(*B*_1_,*B*_2_,*P*) or *F*_2_(*B*_2_,*B*_1_,*P*), so that the parameters for *F*_1_(*B*_1_,*B*_2_,*P*) and *F*_2_(*B*_2_,*B*_1_,*P*) must be bounded to match the observed consumption rates. We adopt this framework of inverse modeling in viewing how alternative energetic conditions imply differences in the underlying model parameters and functions, and how those in turn affect the likelihood of top-down control. For instance, given an observed consumption rate on prey species 1 (*C*_1_), there is an equilibrium prey biomass, *B*_1_*, such that *R_B_*_1_(*B*_1_*)  = *C*_1_. If *B*_1_* is known, one can solve for the parameters of the function *R_B_*_1_(*B*_1_) that satisfy this equality.

### Specified Model

We adopt commonly-used representations for prey growth, prey consumption, predator recruitment, and mortality to explore alternative energetic configurations.

#### Prey growth

We used the logistic model to represent the production function of prey species *j*, *R_Bj_*(*B_j_*):
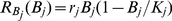
(2)


Because this model is in biomass units, the parameters *r*_j_ denote the maximum ratio of production to biomass for prey species *j*. At equilibrium *R_B_*_1_(*B*_1_) equals *C*_1_ and *R_B_*_2_(*B*_2_) equals *C*_2_ (observed total consumption of prey species 2 by the predator), which leads to two possible prey equilibrium values for prey species j: 
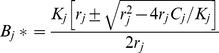
(3)


**Figure 1:**
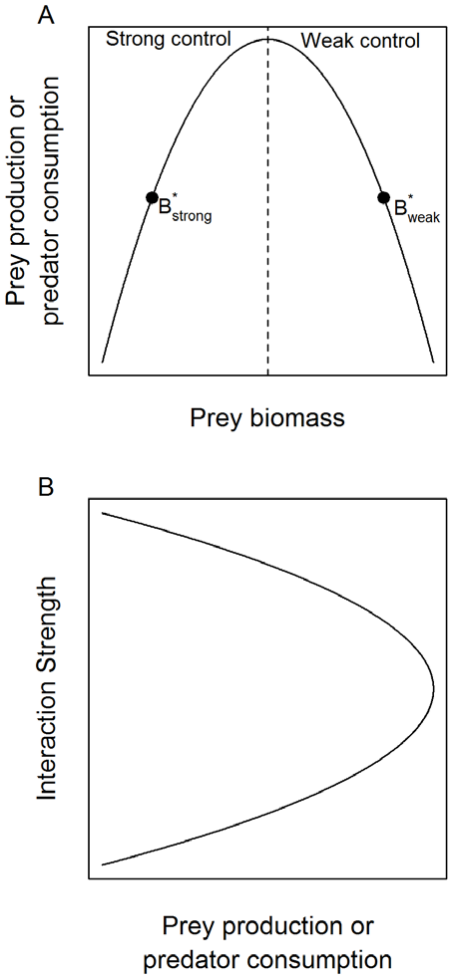
Relationship between predator consumption rate and top-down interaction strength on prey. A typical prey production function, showing two possible equilibrium prey biomasses for a given total observed consumption rate of prey by predators. Model solutions based on the point on the left correspond to strong top-down control, while model solutions based on the point on the right correspond to relatively weak top-down control. Increasing total consumption weakens strong interactions and strengthens weak interactions.

Two points are salient about this relationship. The first is that for a given rate of consumption on prey, there are two possible underlying models: one of which produces strong top-down control, the other produces weak top-down control (Fig. 1a). This relationship is helpful because we can explore model behavior under two alternative underlying models (hereafter, we refer to these as strong- and weak-control), given identical steady state energetics. Second, the magnitude of top-down control for strong interactions (defined as the difference between B_j_* and K_j_) is inversely related to the steady state consumption rate on prey (Fig. 1b).

#### Consumption

We considered both Type I and Type II functional responses to depict the consumption of prey by predators. For the Type I functional response, the predation function *F_j_* for prey species *j* depends only on that species' biomass and predator biomass:
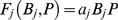
(4)


Because *F_j_*(*B_j_*,*P*) must necessarily equal *C_j_*, and B_j_* is calculated from (3), the attack rate parameter, *a_j_*, is calculated directly from observed predator biomass:
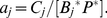
(5)


We used the following form of the multi-species type II functional response:
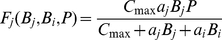
(6)


where *C*_max_ is the maximum possible, per-capita feeding rate of predators, *a_j_* is the rate of effective search of predators on prey *j*. The attack rate parameters can be solved from the observed C_j_*, P* and by estimating prey biomasses *B*_1_* and *B*_2_* from (3):
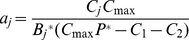
(7)


Thus, the entire system of model parameters can be estimated based on steady state properties.

#### Predator dynamics

Describing predator dynamics requires specifying four functions. The energy assimilation rates are related to the consumption rate on each species. We chose the simplest representation of this process, whereby assimilated energy is a linear function of consumption related through a constant species-independent assimilation efficiency term ( μ). Thus at equilibrium, *G_j_*(*B_j_*,*B_i_*,*P*)  =  μ*F_j_*(*B_j_*,*B_i_*,P) =  μ*C_j_*. Predator recruitment was chosen to be a density dependent Ricker function to ensure that predators had a non-zero equilibrium biomass in the absence of either prey species:
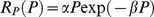
(8)


We parameterized the Ricker function so that it would not contribute to stability calculations described below. Specifically, by setting *β* = 1/P*, we ensured that *dR_P_*(*P*)/*dP* = 0. Lastly, the predator mortality function *M(i)* was chosen as a simple linear function of predator biomass: *M*(*P*) = *mP*.

For any observed P*, *C*_1_ and *C*_2_, the values of the recruitment and mortality function need to be calculated to ensure *dP*/*dt* = 0. Either *m* or α needs to be specified; we chose to always specify *m* and solve for α:
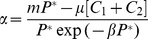
(9)


#### Selecting rate parameters

Yodzis and Innes [25] identified biological constraints on the values of rate parameters used in these models based on predator: prey body size ratios and whether they represented vertebrates or invertebrates and endotherms or ectotherms. We based our model parameterizations by assuming all species are vertebrate ectotherms, however we note that assuming endothermic predators (higher per-capita consumption rates and lower gross assimilation efficiency) does not affect the model's qualitative results (i.e. the exact points at which equilibria switch from being stable to unstable varies depending on the particular parameterization, but qualitatively the patterns hold across a large range of predator-prey pairs). Yodzis and Innes [25] scale all biological rate parameters relative to the maximum production: biomass ratio of the basal resource and scale biomass density units such that *K* of the basal resource equals 1. For simplicity, we assume that *r_1_* = *r_2_* and *K_1_* = *K_2_*. If we assume a predator: prey biomass ratio of 0.05, then *C*_max_ should not greatly exceed 0.8 and *m* should not greatly exceed the maximum production to biomass ratio of the predator population, the latter based on allometric scaling (ca. −0.2). All parameter values used in all scenarios are outlined in Table S1.

### Stability of Energetic Scenarios

We used specific parameterization routines described below to evaluate which energetic conditions were most likely to produce stable, top-down control. Stability has commonly been used as a proxy for the likelihood of the predator-prey system persisting in nature [26]. We used two separate measures to quantify stability. The first is the asymptotic rate at which the system moves from a perturbation back to an equilibrium point (return rate). Negative values imply that the equilibrium is unstable, while small positive values imply slow recovery from a perturbation. While return rate gives a measure of long term behavior after perturbation, reactivity provides an analogous rate assessed immediately following disturbance. Positive values of reactivity imply that some perturbations initially amplify in magnitude. Thus, the most stable systems are those with large (positive) return rates and negative reactivities. Both reactivity and return rate are calculated from the nine partial derivatives of the three dynamic equations in (1) with respect to *B*_1_, *B*_2_ and *P* [27], [28].

### Description of Energetic Scenarios

**Figure 2:**
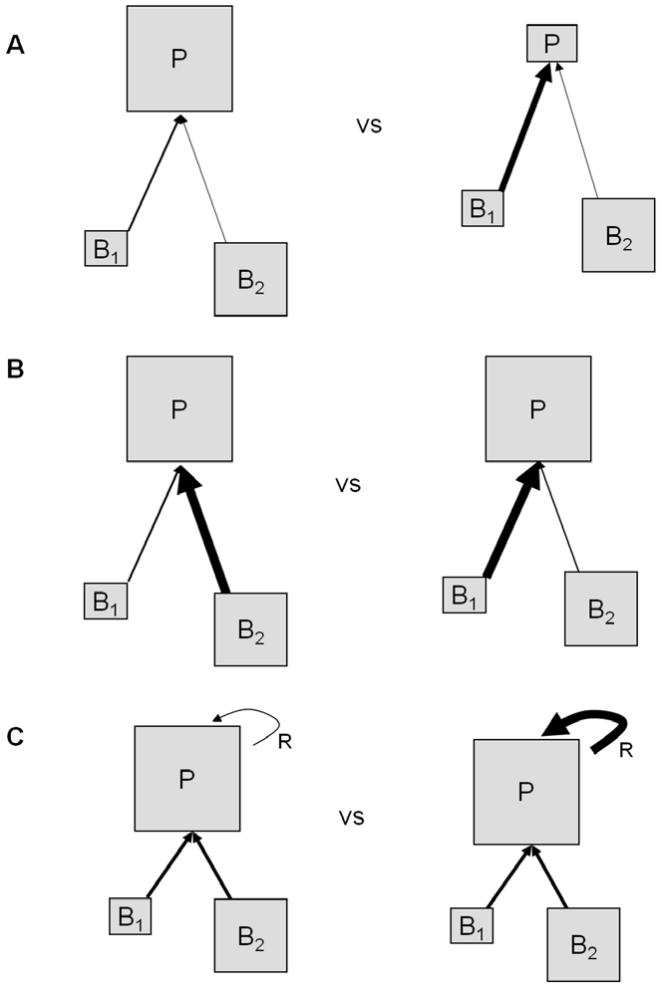
Schematic of predator energetic configurations. The size of the boxes surrounded by solid lines (*B* and *P*) represent their relative biomass in different configurations. Arrows indicate the direction and relative magnitude of energy flux. The scenarios are as follows: per capita consumption (A), consumption of preferred prey versus other prey (B), and contribution of recruitment, *R* (C).

We considered three alternative energetic conditions that might have bearing on the stability of predator-prey systems. In all scenarios we assume a strong top-down control of the predator on prey species 1 (left-hand equilibrium in Figure 1a). The first scenario evaluated predator per-capita consumption (Fig. 2a). In this first scenario, we specified a constant steady state total consumption rate, and then consider model stability under a suite of alternative predator biomasses and per-capita consumption rates (*p_c_*). Our second energetic scenario explored the composition of prey consumption. Predators might be generalists or their diets may be dominated by a single prey species (Fig. 2b). To evaluate the impact of prey diet composition on stability, we specified the fraction of predators' diet that was composed of species 1 (*p_D_*), predator biomass and held total predator consumption constant, and adjusted *C_1_* and *C_2_* according to the value of *p_D_*. For our third scenario, we considered the relative importance of consumption and recruitment in the predators' energy balance; some populations might receive little biomass (energy) from recruitment of offspring (for example, species that undergo ontogenetic diet shifts), while for other predators, this energy source may be substantial (Fig. 2c). To explore these effects, *C*_1_, *C*_2_ and P* were held constant, and we adjusted the proportion of energy input derived from recruitment, denoted *p_r_*. For each *p_r_*, we calculated total recruitment, and the predator mortality rate that would therefore be needed to achieve steady state.

## Results

### Scenario 1: Per-capita consumption rates

**Figure 3:**
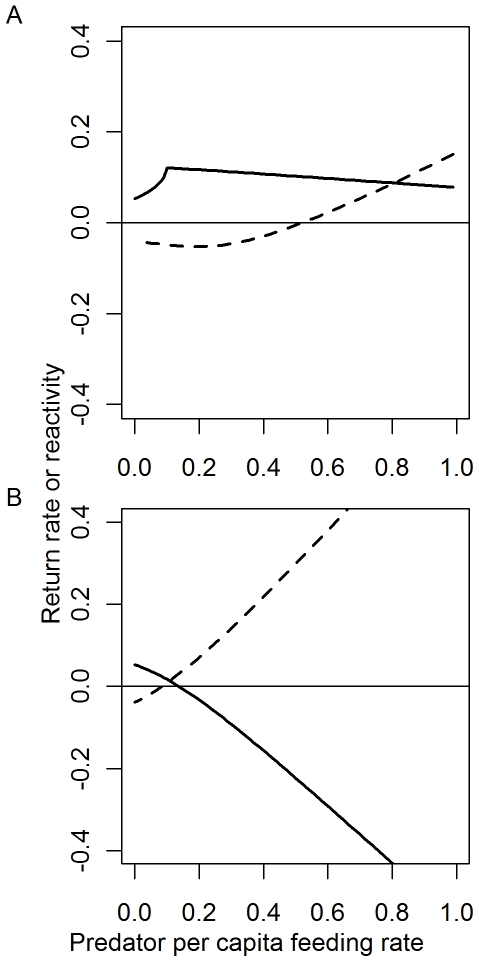
Effect of per-capita feeding rate on model stability. Total equilibrium consumption on each prey is held constant, but per-capita feeding rate (*p_c_*) and predator biomass is adjusted, *C_1_* = *C_2_* = 0.05; µ = 0.1, *m* = 0.2. The solid line indicates return rate and the dashed line indicates reactivity. (A) Predator consumption is modeled with a non-saturating functional response. (B) Predator consumption is modeled with a saturating functional response, *C*_max_ = 0.8.

We first considered the scenario when predators exert strong control on prey species 1 but weak control on prey species 2 and there is no satiation in the predator's functional response. Regardless of the assumed values of other parameters, reactivity is always greatest for high per-capita consumption rates (Fig. 3). Return rate showed a more complex relationship with per-capita consumption rate, and this relationship depended on the assumed value for the predator mortality rate (*m*). When *m* is set to the upper bound of the biologically plausible range (ca. 0.2, based on presumed predator: prey body size ratios), return rate initially increases with per-capita consumption, eventually reaches a threshold value, and then declines. Over the range of parameter values where return rates increase, model equilibria exhibit no oscillatory behavior and strengthening per-capita effects of predators on prey allows systems to return to equilibrium more quickly because prey populations become more sensitive to changes in predator population sizes. Eventually this interaction becomes too strong and the system begins to destabilize in the form of oscillatory behavior (this is demarcated by the abrupt shift in the slope of the plot of return rate vs. per-capita consumption, Fig. 3a).

The destabilizing effects of high per-capita consumption are stronger when predation is represented by a saturating functional response (maximum consumption rate  = 0.8, Fig. 3b). Reactivity increases sharply with per-capita consumption rate over the entire parameter space considered. Regardless of the values of the other parameters, return rate declines with increasing per-capita consumption rate producing negative (i.e. unstable) values over much of the range. These relationships reflect the fact that the well-known destabilizing effects of saturating functional responses are greatest when predators are feeding near their asymptotic rate.

### Scenario 2: Diet composition

**Figure 4:**
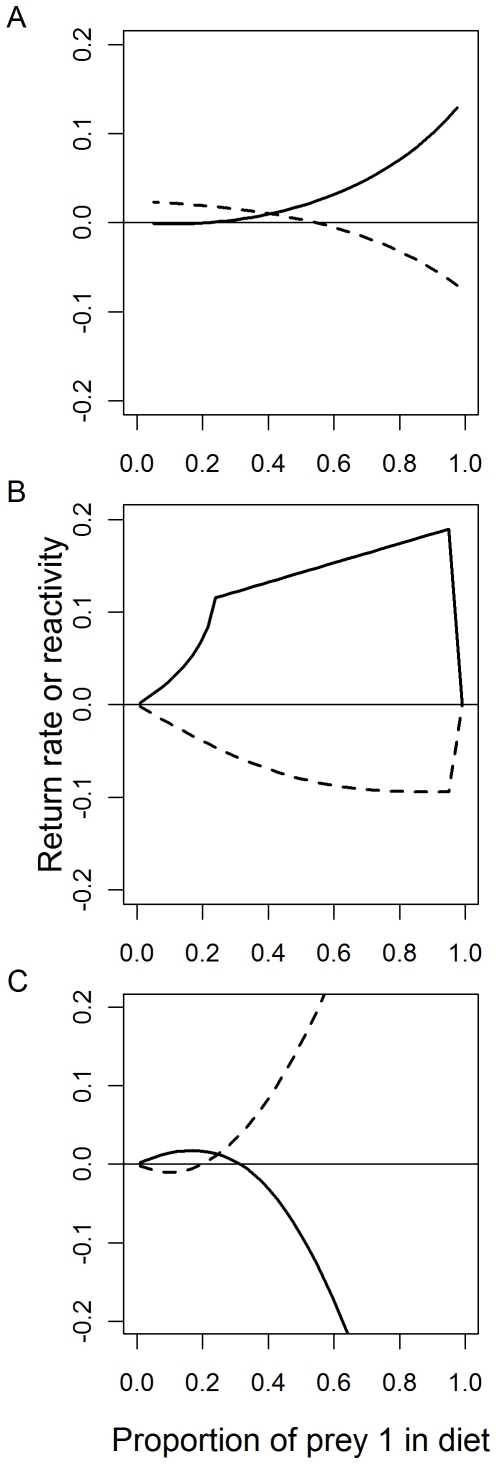
Model stability vs. the proportion of predator diet comprised of top-down controlled prey species. (A) Total predator consumption is held constant, so that the equilibrium consumption of predators on each prey species is variable. Predator consumption is modeled with a saturating functional response. (B) Same as A, but the parameters of each prey species production function is adjusted so that interaction strengths are held constant. Satiation is modeled as a non-saturating functional response. (C) Same as B, but with a saturating functional response. Model parameters: *m* = 0.2; µ = 0.1; *C*_1_ + *C*_2_ = 0.2; *C*_max_ (if used) = 0.8; P* = 1, lines are as defined in Figure 1 A and B.

In this scenario we asked whether the stability of the predator-prey system is enhanced or diminished by changing the relative importance of two alternative prey. Throughout this scenario, we assume strong control on prey species 1, and denote the proportion of total consumption comprised of this species as *p_D_*. When the second prey species is under weak control, we find that reactivity declines and return rate increases as the contribution of prey species 1 to the diet is increased, regardless of whether predation is modeled with or without satiation (Fig. 4a). This result is largely due to the fact that changing the predator's diet changes the strength of top-down control on the two prey species. That is, increasing *p_D_* increases consumption on prey species 1 while decreasing consumption on prey species 2. Because of the different marginal effects of consumption that occur when equilibrium abundance is on the left or right hand side of the curve in Figure 1a, these changes have different effects on the strength of interaction of the two species. Because prey species 1 is presumed to be strongly top-down controlled (left-side of function, Figure 1a), increasing *p_D_* enhances consumption and therefore weakens the interaction strength on this species (Figure 1b). At the same time, increasing *p_D_* decreases consumption on prey 2, and because this species is on the right-hand side of the function (Figure 1a,b) this causes the interaction strength to be weakened as well. Thus, the effect of increasing *p_D_* is to weaken the predator's interaction strength on both prey species which increases model stability as measured by reactivity and return rate. In contrast, if the predator exerts strong top-down control over both prey species, increasing *p_D_* decreases the strength of top-down control for prey 1 and simultaneously increases the strength of top-down control for prey 2 to maintain constant total consumption.

Another way to examine the effect of shifting predator diet on stability is to presume fixed interaction strength of predators on each prey species. This can be done by fixing the equilibrium biomasses of prey species 1 and 2 at a specified value to produce a strong or weak interaction and adjusting the maximum production rate, *r*, to satisfy the equilibrium condition:
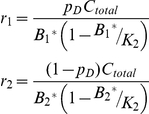



With a non-saturating functional response, reactivity was always negative (Fig. 4b) and all perturbations from equilibrium result in the system initially moving back towards equilibrium. Return rates generally increased with increasing *p_D_*, however when *p_D_* approached 1 (*p_D_*>0.95), return rates declined rapidly. This result occurs because extremely low attack rates on prey 2 decouple prey 2′s dynamics from the predator. In general, both measures of stability point to more stable outcomes in our model when a dominant part of the predators' diet is composed of prey items that are strongly controlled by predation. The mechanism behind these outcomes in our model is that increasing *p_D_* leads to increased consumption of (and therefore higher attack rate on) prey species 1, the species that is most strongly controlled by predation. One can therefore think of the scenarios when *p_D_* is large as one in which predators have a high attack rate on a top-down controlled prey species and a low attack rate on another prey species that is largely regulated by density dependent resource limitation. Setting *p_D_* to high values requires that *r_1_* be quite large. As a result, small reductions in predator biomass result in rapid increases in prey biomass, which in turn fuels the recovery of the predator back to equilibrium. In contrast, when *p_D_* is low, both the attack rate on prey 1 and the maximum production rate are also low, such that there is little response of prey species 1 to a small perturbation in predator biomasses.

The impact of predator diet composition on model stability is completely reversed when predation is constrained by handling time or satiation (Figure 4c). Reactivity is initially negative for small values of *p_D_*, but increases rapidly as the diet fraction consisting of prey 1 increases. More notably, return rate is positive for only small values of *p_D_* and becomes negative (unstable equilibrium) at low to intermediate values of *p_D_*. The response of the model dynamics to *p_D_* is driven largely by the instability in the linkage between the predator and prey1. When consumption rates on prey 1 are low, the stabilizing density-dependent effects in the logistic function overwhelm the destabilizing effects of inverse density dependence in the functional response. As diet composition shifts to include more preferred prey, this produces an increase in total consumption of prey 1 (while maintaining a constant total predation rate of predators relative to their maximum rate), so that the destabilizing effects of predation become stronger than the stabilizing effects.

### Scenario 3: Contributions of consumption and recruitment

**Figure 5:**
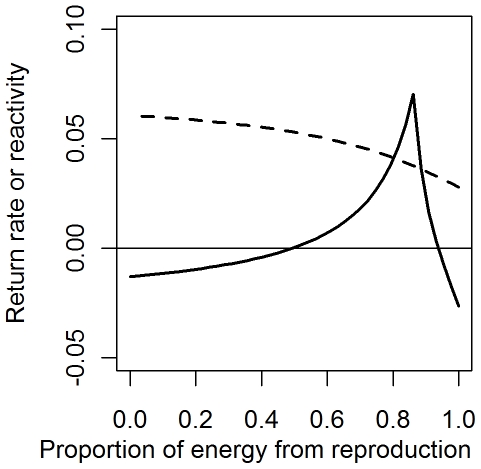
Effect of the proportion of predators ' **energy derived from reproduction (** ***p_r_*** **) on model stability.** Reactivity of an unstable model cannot be made negative by increasing the proportion of energy from recruitment, but the return rate can be increased. The stabilizing effects of recruitment-derived energy erode when recruitment dominates energy inputs to predators. Model parameters: P* = 0.75; *C*_1_ = *C*_2_ = 0.1.

Here we asked whether a system that is otherwise unstable (positive reactivity and negative return rate) might be stabilized by adjusting the relative importance of consumption and recruited offspring. We considered the most unstable case examined thus far: a saturating functional response, with high per-capita consumption rates of prey and low predator biomass. Total recruitment was adjusted to be a fraction *p_r_* of the total energy input. We solved for the parameter α: 


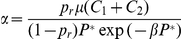
 and to satisfy equilibrium, *m = *αexp(−βP*)*+* µ(C_1_+C_2_)/P*. In general, increasing the proportion of energy derived from recruitment improves stability with respect to both reactivity and return time (Fig. 5). Specifically, oscillatory model behavior decreases as *p_r_* increases until density dependence in recruitment becomes so high that it destabilizes the system. Changing *p_r_* only affects one component of the stability calculations, the derivative of the predator dynamic equation with respect to predator biomass. When *p_r_* is set low, recruitment and mortality are low to satisfy equilibrium conditions. Because we wanted to ensure that the choice of *P* had no bearing on the model stability through its affect on recruitment, parameters of the recruitment function were chosen so that its slope with respect to predator biomass is 0 when at equilibrium (it neither contributes to, nor undermines the model stability). In biological terms, this means that for very small changes in *P*, there is little change in predator recruitment.

The stabilizing effects from increasing the relative contribution of recruitment are not universal. If recruitment becomes too large of a source of energy compared to consumption, the predator dynamics become decoupled from the dynamics of the underlying prey. This means a drop of prey species 1 biomass will produce only modest reduction in predator biomass, which in turns ensures a continuing high consumption pressure on prey species 1. In these cases, the model can become destabilized by causing prey species 1 to either become extinct or move to a new equilibrium with a reduced top-down control from predators (depending on the nature of the perturbation). A relevant biological example is an adult fish population where most of the biomass growth came from new individuals recruiting from lower age classes, rather than from somatic growth due to prey consumption in the adult stage. Small changes in the strongly controlled prey populations will not be felt by the predator population in this case, because its dynamics would not be driven by consumption.

## Discussion

Here we used a theoretical model to develop rules of thumb to identify energetic conditions that might predispose strong top-down control. Specifically, we used a simple and tractable model, parameterized with biologically plausible rates, to evaluate hypothetical energetic conditions to determine what they imply about the stability of strong top-down predator control. Our model suggests that at the population level the strongest possible interactions occur when total energy flow from prey to predators is relatively low. This somewhat counter-intuitive result is understood by recognizing that when predators exert strong control on prey, they can deplete prey to very low levels so that total consumption of prey by predators is low. Moreover, these energetic configurations tended to be stable compared to configurations with higher rates of energy flow. However, the model also revealed that this finding was dependent on the shape of the functional response. High diet fractions were most stable for non-saturating functional responses while low diet fractions were most stable for saturating functional responses. Finally, our model showed that high rates of energy flux from recruited offspring, themselves dependent on different prey resources, can stabilize otherwise very unstable predator-prey linkages. These heuristics can guide decisions about future data collection that can then refine hypotheses about key ecosystem players and interactions.

Recent work by Berlow et al. [22] predicted interaction strengths based on biomass and body-size ratios of predators and prey. They, like us, found that low prey biomass was associated with the strongest population interaction strengths. Notably, they also found that functional response type did not affect their results, while the form of the functional response played a key role in stability in our study. However, the functional responses Berlow et al. considered were a range between Type II and Type III responses, and did not include a linear functional response that we considered here.

Overall, this modeling exercise demonstrated that many factors act to confound simple generalized predictions about the stability of particular predator-prey configurations. Attack rates and per capita growth rates were especially important in dictating how populations responded to perturbations. Depending on these values, a wide range of per capita consumption rates and diet compositions may give rise to stable top-down control by a predator. Moreover, we frequently observed reactivity and return rate behaving in opposite directions, i.e. reactivity tending towards less stable but return rate tending towards more stable interactions. For instance, predator and prey populations that are quite decoupled are not reactive, but may take longer to return to equilibrium after perturbation because attack rates and growth rates are not high enough to fuel recovery. In situations with high attack rates and growth rates, predator and prey populations are more tightly coupled, leading to faster responses and quicker return rates, but more reactive systems. Only intermediate levels of energetic coupling tended to consistently provide low reactivity and high return rates.

Simple models can build intuition about the relationships between observed phenomena and the processes that lead to them. By ignoring many of the details of biological reality and focusing on three species, we explicitly described relationships between energetic processes, top-down control, and equilibrium stability. Further, processes captured in our model (e.g. consumption of multiple prey and recruitment) were flexible enough to reasonably describe important processes for many species and systems. Biological realities often serve to reduce direct effects and dampen oscillations, and diffuse effects are a common characteristic of many systems [21], [29], [30]. Therefore, some of the equilibria predicted by our models to be unstable may indeed be stable when taken in the context of their larger food web. In this way, we see the model predictions as an initial, rather than final, step in exploring the possibility of top-down control in any given system.

Whether our heuristic model's predictions can accurately predict top-down control remains to be seen. Because no standard exists for reporting interaction strengths, conducting a complete meta-analysis is difficult – however, we can do a retrospective analysis of well-studied systems and ask whether our model is consistent with observed interaction strengths, given some energetic information about the predator-prey pair. We describe three particularly well-known predator-prey systems and how our simple predictions apply to them: bass in temperate lakes, grey wolves on Isle Royale National Park, and cod in the Atlantic.

A classic example of strong top-down control of predators on prey is bass predation on small forage fish in temperate lakes [31]. Bass and most piscivorous fishes undergo ontogenetic diet shifts, with young fish feeding on zooplankton and aquatic insect larvae [32]. Adult fish feed on both small prey fish (e.g. minnows) as well as benthic invertebrates. About 30 percent of bass diets are made up of these forage fish, while the remainder are benthic invertebrates [33]. It has been suggested that the subsidy of invertebrates from benthic habitats is what allows, and even encourages, bass to become heavy predators on the forage fish resource; this result has also been described in other piscivores [33], [34].

As a second example, we consider wolf and moose in Isle Royale National park. Data on their abundance dates back more than 40 years. Moose are the primary prey of wolves in this system (approximately 90 percent of total diet by biomass [35]), and therefore our model would again predict that this interaction should be weak, or strong and relatively unstable. Abundance data indicate that the time series may support long term oscillations [36], suggesting that the effect of predators on prey is greater than the intra-specific density dependent effects in the predator and prey populations. These oscillating populations may provide an example of a resilient but reactive equilibrium that persists through time, and recently it has been suggested that disease in the predator population plays a role in modulating the switches between predator and prey dominated states [37].

Finally, we consider an example in which harvest of predators (cod in the North Atlantic) released prey from predation and may have ultimately caused an ecosystem state shift. Since cod stocks collapsed, two prey species of shrimp and crab have surged in abundance [5], [38]. Although pandalid shrimp contribute only 3 to 9 percent of cod diet, Worm and Myers found evidence for top-down control of shrimp by cod. Our modeling framework would suggest that this relatively small energy flux is more likely to give rise to top-down control than a large energy flux. The small proportion of total predator diet alone would lead us to believe this interaction has the potential to be strong, stable, and nonreactive. However, since all age classes of cod could consume these small prey, predation would not be stabilized by recruitment in the predator population. Given this lack of stabilizing forces from the predator and relatively high reproductive rates of pandalid shrimp, we would predict this interaction to be reactive, which may provide an explanation for the dramatic nature of the ecosystem shift.

These three examples, while providing some additional context for predictions about top-down control, are certainly not exhaustive. An extension of this work would be a formal review of published instances of top-down control coupled with explorations of energetic processes in each of the systems. The methods described in this paper are a step in the direction of trying to identify important predator-prey links in food webs. Predation is not the only force at work structuring ecosystems, but bettering our understanding and predictive abilities with regards to its dynamic role will help to decrease the number of “surprises” [39] that occur when harvest and recovery strategies produce unexpected outcomes.

## Supporting Information

Table S1 Model parameter values for energetic scenarios
(DOC)

## References

[1] Clark JS, Carpenter SR, Barber M, Collins S, Dobson A (2001). Ecological forecasts: An emerging imperative.. Science.

[2] Crooks KR, Soule ME (1999). Mesopredator release and avifaunal extinctions in a fragmented system.. Nature.

[3] Terborgh J, Lopez L, Nunez P, Rao M, Shahabuddin G (2001). Ecological meltdown in predator-free forest fragments.. Science.

[4] Treves A, Karanth KU (2003). Human-carnivore conflict and perspectives on carnivore management worldwide.. Conserv Biol.

[5] Worm B, Myers RA (2003). Meta-analysis of cod-shrimp interactions reveals top-down control in oceanic food webs.. Ecology.

[6] Sibert J, Hampton J, Kleiber P, Maunder M (2006). Biomass, size, and trophic status of top predators in the Pacific Ocean.. Science.

[7] Borer ET, Seabloom EW, Shurin JB, Anderson KE, Blanchette CA (2005). What determines the strength of a trophic cascade?. Ecology.

[8] Shurin JB, Seabloom EW (2005). The strength of trophic cascades across ecosystems: predictions from allometry and energetics.. J Anim Ecol.

[9] Paine RT (1980). Food webs: linkage, interaction strength, and community infrastructure.. J Anim Ecol.

[10] Wootton JT (1992). Indirect Effects, Prey Susceptibility, and Habitat Selection - Impacts of Birds on Limpets and Algae.. Ecology.

[11] Power ME, Tilman D, Estes JA, Menge BA, Bond WJ (1996). Challenges in the quest for keystones.. Bioscience.

[12] Schmitz OJ (2004). Perturbation and abrupt shift in trophic control of biodiversity and productivity.. Ecol Lett.

[13] Ives AR, Dennis B, Cottingham KL, Carpenter SR (2003). Estimating community stability and ecological interactions from time-series data.. Ecol Monogr.

[14] Wootton JT (1994). Predicting Direct and Indirect Effects - an Integrated Approach Using Experiments and Path-Analysis.. Ecology.

[15] DeAngelis DL (1992). Dynamics of Nutrient Cycling and Food Webs..

[16] Polis GA (1991). Complex Trophic Interactions in Deserts - an Empirical Critique of Food-Web Theory.. Am Nat.

[17] Polis GA, Strong DR (1996). Food web complexity and dynamics.. Am Nat.

[18] Rasero M, Gonzalez AF, Castro BG, Guerra A (1996). Predatory relationships of two sympatric squid, Todaropsis eblanae and Illex coindetii (Cephalopoda: Ommastrephidae) in Galician waters.. J Mar Biol Assoc UK.

[19] Holling CS, Watt KEF (1966). The strategy of building models of complex ecological systems.. Systems Analysis and Simulation in Ecology.

[20] Rip JMK, McCann KS, Lynn DH, Fawcett S (2010). An experimental test of a fundamental food web motif.. Proc R Soc Lond, Ser B: Biol Sci.

[21] McCann K, Hastings A, Huxel GR (1998). Weak trophic interactions and the balance of nature.. Nature.

[22] Berlow EL, Dunne JA, Martinez ND, Stark PB, Williams RJ (2009). Simple prediction of interaction strengths in complex food webs.. Proc Natl Acad Sci USA.

[23] Walters C, Christensen V, Pauly D (1997). Structuring dynamic models of exploited ecosystems from trophic mass-balance assessments.. Rev Fish Biol Fish.

[24] Berlow EL, Neutel AM, Cohen JE, de Ruiter PC, Ebenman B (2004). Interaction strengths in food webs: issues and opportunities.. J Anim Ecol.

[25] Yodzis P, Innes S (1992). Body size and consumer-resource dynamics.. Am Nat.

[26] Pimm SL, Lawton JH (1977). Number Of Trophic Levels In Ecological Communities.. Nature.

[27] May RM, Princeton (1973). Stability and Complexity in Model Ecosystems.. N.J.: Princeton University Press.

[28] Neubert MG, Caswell H (1997). Alternatives to resilience for measuring the responses of ecological systems to perturbations.. Ecology.

[29] Yodzis P (2000). Diffuse effects in food webs.. Ecology.

[30] Berlow EL (1999). Strong effects of weak interactions in ecological communities.. Nature.

[31] Carpenter SR, Cole JJ, Hodgson JR, Kitchell JF, Pace ML (2001). Trophic cascades, nutrients, and lake productivity: whole-lake experiments.. Ecol Monogr.

[32] Werner EE, Gilliam JF (1984). The ontogenetic niche and species interactions in size-structured populations.. Annu Rev Ecol Syst.

[33] Vander Zanden MJ, Vadeboncoeur Y (2002). Fishes as integrators of benthic and pelagic food webs in lakes.. Ecology.

[34] Vander Zanden MJ, Essington TE, Vadeboncoeur Y (2005). Is pelagic top-down control in lakes augmented by benthic energy pathways?. Can J Fish Aquat Sci.

[35] Thurber JM, Peterson RO (1993). Effects of population-density and pack size on the foraging ecology of gray wolves.. J Mammal.

[36] Peterson RO, Thomas NJ, Thurber JM, Vucetich JA, Waite TA (1998). Population limitation and the wolves of Isle Royale.. J Mammal.

[37] Wilmers CC, Post E, Peterson RO, Vucetich JA (2006). Predator disease out-break modulates top-down, bottom-up and climatic effects on herbivore population dynamics.. Ecol Lett.

[38] Frank KT, Petrie B, Choi JS, Leggett WC (2005). Trophic cascades in a formerly cod-dominated ecosystem.. Science.

[39] Doak DF, Estes JA, Halpern BS, Jacob U, Lindberg DR (2008). Understanding and predicting ecological dynamics: Are major surprises inevitable?. Ecology.

