# Supplementary material for: Energetic Conditions Promoting Top-Down Control of Prey by Predators
Source: PLoS One. 2011 Dec 27;6(12):e29723. doi: 10.1371/journal.pone.0029723 (PMC3246494; doi:10.1371/journal.pone.0029723)
Supplement: Table S1 — Model parameter values for energetic scenarios (DOC) [file pone.0029723.s001.doc]

**Table S1**. Model parameter values for energetic scenarios

| Parameter | Scenario 1 | Scenario 2a | Scenario 2b | Scenario 3 |
| --- | --- | --- | --- | --- |
| *r_1_* | 1 | 1 | Variable | 1 |
| *r_2_* | 1 | 1 | Variable | 1 |
| *K_1_* | 1 | 1 | 1 | 1 |
| *K_2_* | 1 | 1 | 1 | 1 |
| *B_1_** | 0.05 | Variable | 0.11 | 0.11 |
| *B_2_** | 0.95 | Variable | 0.89 | 0.89 |
| *C_max_* | 0.8 | 0.8 | 0.8 | 0.8 |
| *C_total_* | 0.1 | 0.2 | 0.2 | 0.2 |
| *C_1_* | 0.05 | Variable | Variable | 0.1 |
| *C_2_* | 0.05 | Variable | Variable | 0.1 |
| *P** | Variable | 1 | 1 | 0.75 |
| *μ* | 0.1 | 0.1 | 0.1 | 0.1 |
| *m* | 0.2 | 0.2 | 0.2 | Variable |
| *p_c_* | Variable | --- | --- | --- |
| *p_D_* | --- | Variable | Variable | --- |
| *p_r_* | --- | --- | --- | Variable |
